# Pharmacogenomics of Targeted Agents for Personalization of Colorectal Cancer Treatment

**DOI:** 10.3390/ijms18071522

**Published:** 2017-07-14

**Authors:** Alessia Bignucolo, Elena De Mattia, Erika Cecchin, Rossana Roncato, Giuseppe Toffoli

**Affiliations:** Clinical and Experimental Pharmacology, CRO-National Cancer Institute, via Franco Gallini 2, 33081 Aviano (PN), Italy; alessia.bignucolo@cro.it (A.B.); ececchin@cro.it (E.C.); rroncato@cro.it (R.R.); gtoffoli@cro.it (G.T.)

**Keywords:** targeted agents, anti-EGFR agents, antiangiogenic molecules, Vascular Endothelial Growth Factor (VEGF), pharmacogenomics, somatic mutation, Rat Sarcoma Oncogene (RAS), inflammation, metastatic colorectal cancer

## Abstract

The use of targeted agents in the treatment of metastatic colorectal cancer (CRC) has improved patient outcomes. Anti-epidermal growth factor receptor (anti-EGFR) agents (cetuximab and panitumumab) and antiangiogenic molecules (bevacizumab, regorafeninb, ramucirumab, and aflibercept) have been successfully integrated into clinical practice. Other drugs have been designed to target additional deregulated pathways in CRC, such as MAPK (mitogen-activated protein kinase)/PI3K-AKT (phosphatidylinositol-3-kinase-AKT serine/threonine kinase)/mTOR (mammalian target of rapamycin), HER-2 and 3 ( human epidermal growth factor receptor-2 and -3), and BRAF. A major issue with targeted treatment is early identification of patients with primary or secondary drug resistance. Pharmacogenomic research has demonstrated its value in this field, highlighting some tumor mutations that could discriminate responders from non-responders. The tumor genetic profile of the RAS/RAF pathway is needed before treatment with anti-EGFR agents; mutations in EGFR pathway genes have also been explored in relation to antiangiogenic molecules although further data are required prior to their integration into clinical practice. The introduction of immunotherapy has paved the way for a new generation of predictive markers, including genome-wide assessment of the tumor landscape. Furthermore, the development of next generation sequencing technology and non-invasive approaches to analyze circulating tumor DNA will make real-time monitoring of the tumor pharmacogenomic markers possible in the clinical routine, rendering precision medicine available to every patient.

## 1. Introduction

Colorectal cancer (CRC) is a heterogeneous disease with an incidence of 9.7%, representing the third most frequent cancer worldwide in both sexes. The number of new CRC cases worldwide is estimated to increase from 1.4 million (in 2012) to 2.4 million by 2035. A small proportion of CRC cases appear to be hereditary, whereas 90–95% of cases are sporadic CRC with a high capacity to accumulate mutations (IARC-Globocan [1]). Despite important advances in the treatment of CRC, it remains one of the deadliest cancer, with an increasing incidence in non-Western countries [2,3]. The choice of treatment depends on TNM (Tumor-Lymph Nodes-Metastasis) staging, which refers to local invasion, lymph node involvement, and degree of metastasis. Complete surgical removal of the primary tumor and regional lymph nodes is the main treatment for CRC, especially in the earliest stages (I and II). In stage III colon cancer with ganglion involvement, adjuvant chemotherapy is recommended in addition to surgical intervention in order to reduce the likelihood of relapse and increase healing. The use of adjuvant treatment in stage II cases is more controversial because of the different risk of recurrence [4] and considered an acceptable treatment option only for high-risk stage II patients [5]. Systemic chemotherapy is often the main treatment for advanced CRC and is preceded, in some cases, by surgery at sites of metastasis. In the last two decades, the treatment of CRC, especially advanced disease, has experienced significant progress with the introduction of novel target agents used alone or, more frequently, in combination with conventional chemotherapeutics, improving CRC patient management. Despite surgery and chemotherapy based on traditional cytotoxic agents (i.e., fluoropyrimidines, irinotecan, and oxaliplatin) remaining the cornerstone CRC therapy for more than 10 years now, the Food and Drug Administration (FDA) and European Medicines Agency (EMA) have approved new molecules that improve the therapeutic effectiveness, overall survival (OS), and progression-free survival (PFS). In particular, research efforts have focused on novel agents targeting tumor angiogenic activity, cell growth, and migration in metastatic CRC (mCRC). The use of molecules targeting the epidermal growth factor (EGF) (i.e., cetuximab, panitumumab) and vessel epidermal growth factor (VEGF) pathways (i.e., bevacizumab, ziv-aflibercept, regorafenib, and ramucirumab) have been integrated into clinical practice. Anti-EGF receptor (EGFR) monoclonal antibodies (mAbs) are employed for the treatment of mCRC in combination with chemotherapy Fluorouracil, Leucovorin and Oxaliplatin (FOLFOX), Fluorouracil, Leucovorin and Irinotecan (FOLFIRI), Irinotecan, Leucovorin and Fluorouracil (IFL), as first- or second-line therapy [6]. Several ongoing trials are currently assessing the effectiveness of new target agents in order to provide new options for the management of mCRC. Trastuzumab, which is usually administered in breast cancer, is of particular interest due to its recent utilization in HER-2-positive CRC, providing an alternative target for blocking tumor growth. In addition, a combination of two cytotoxic agents (trifluridine plus tipiracil; marketed as Lonsurf, TAS-102) has been approved [7] for second-line treatment of mCRC refractory to 5-FU, irinotecan, and oxaliplatin and in salvage treatment after progression based on the RECOURSE phase III trial (NCT01607957) [8]. A large proportion of anticancer research has been dedicated to immunotherapy since the first half of the twentieth century. Oncological immunotherapy exploits the presentation of tumoral antigens on the cancer cell surface. However, no immunological therapy has been approved for CRC, though significant efforts are ongoing to improve the efficacy of cancer vaccines and their safety profile. Several clinical trials are currently ongoing with the aim to implement immunotherapy for mCRC into clinical practice [9].

Despite recent improvements, the management of patients with mCRC is still difficult due to the significant inter-individual differences in the therapeutic response profile observed in clinical practice. Pharmacogenomics has been applied in recent years to the personalization of CRC treatment [10], and many research efforts have focused on defining the contribution of tumor genetic variants to the variability in outcomes of targeted agent-based therapy. However, regardless of the large body of published data, only a few biomarkers have been identified and validated for use in clinical diagnosis (e.g., Kirsten Rat Sarcoma Viral Oncogene Homolog [KRAS] status for cetuximab administration). In this review, we focus on tumor mutations that have emerged as potential predictive markers of targeted therapy outcome to better understand their real clinical utility in identifying subsets of patients most likely to benefit from the administration of these novel targeted molecules.

## 2. Pharmacogenomics of Approved Molecules Targeting the VEGF and EGF Pathways

Metastatic colorectal disease has achieved a certain improvement in terms of survival and outcome benefit with the introduction of novel targeted molecules to current practice. Yet, a considerable proportion of patients still experience disease progression after a short treatment period and seem to not respond to the therapies, probably due to the onset of resistance mechanisms and the overlap of alternative pathways downstream of the blockade point. Thus, efforts are currently being made to identify somatic genetic variants that explain the diverse response to treatments and identify validated biomarkers to be introduced into clinical practice in order to identify subsets of patients who may benefit more from targeted therapies (Table 1).

**Figure 1:**
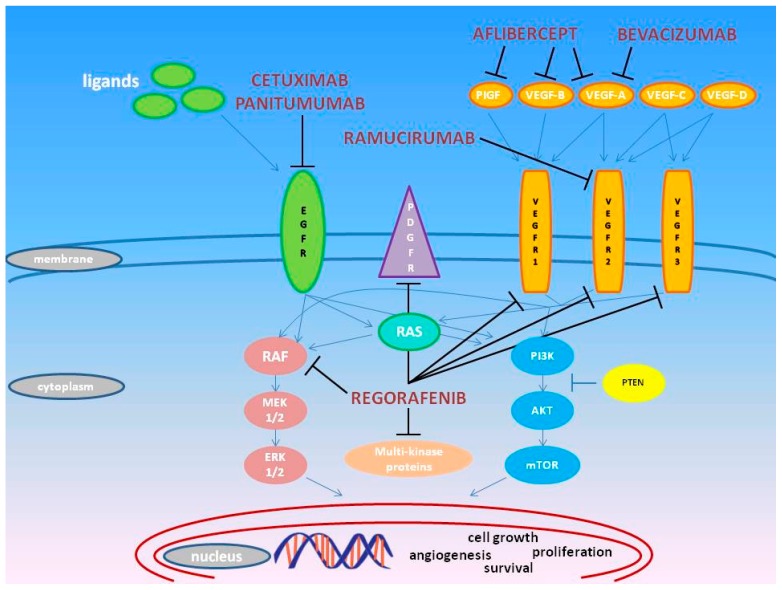
Molecular targets of the approved anti-EGFR and antiangiogenic agents (T arrows) in the EGF and VEGF molecular cascade (blue arrows).

Some targeted molecules (e.g., bevacizumab, cetuximab, panitumumab) have entered clinical practice as standard treatments both in combination with chemotherapy or as single agents for the treatment of mCRC (Figure 1).

### 2.1. Bevacizumab 

Bevacizumab (Avastin®) is a recombinant humanized mAb approved by the FDA in 2004 for the first-line treatment of mCRC in association with chemotherapy. The drug selectively targets VEGF-A, inhibiting its association with the VEGF receptor (VEGFR) and interfering with the angiogenic signaling cascade responsible for tumoral cell proliferation, migration, angiogenic activity, and inhibition of apoptosis [31]. Genetic variability within VEGF-dependent and -independent pathways has been suggested to be related to the response to bevacizumab and progression of metastatic disease [10]. However, the most available data are related to the association between germline genetic polymorphisms and patient outcome; the role of somatic mutations in predicting BV efficacy has been less investigated and only few preliminary data have been published in recent years. While the VEGF-A 5’ untranslated region (UTR) and promoter region has been the major target of investigations focusing on inherent variations, studies on somatic mutations have looked mainly into RAS/RAF/PIK3CA pathway, given the well-known association between activation of KRAS signaling and angiogenesis [11]. Interestingly, Fiala et al., in a large cohort of 404 mCRC Caucasian patients receiving BV-containing treatment, observed that *KRAS* G12A/V mutation type is a significant predictor of shorter PFS and OS respect to *KRAS* wild-type and other *KRAS* mutations [11]. Another investigation, performed on a similar study population in order to compare the FOLFOXIRI plus BV versus FOLFIRI plus BV as first-line treatment of mCRC patients, showed that RAS- and *BRAF*-mutation-positive subgroups have an inferior OS and PFS with respect to *RAS* and *BRAF* wild-type subgroup; in a stratify analysis according to FOLFOXIRI- or FOLFIRI-plus BV regimen, treatment effect was not significantly different across molecular subgroups [12]. The detrimental impact of mutations in EGFR pathway genes on BV efficacy was also reported in Asian mCRC patients. Nakayama et al., evaluating the significance of *RAS/PIK3CA/BRAF* tumor mutations in patients receiving first-line BV-containing treatment, showed that mutant-type tumors have a lower objective response rate (ORR) respect to wild-type tumors and that these differences are greater when considering only *KRAS* exon 2 mutations rather than all *RAS (KRAS* and *NRAS)/PIK3CA/BRAF* mutations. Moreover, in multivariate analysis *RAS* and *BRAF* mutations, together with clinico-pathological parameters, were independent negative factors for disease progression. Even if these differences did not reach the statistically significant level, probably due to the relatively small effect of *RAS* mutations and the rarity of *BRAF* mutations, *RAS/PIK3CA/BRAF* status appeared to be a promising candidate to help in identifying tumors that will respond to the anti-VEGF drug [13]. Another work was not able to found an association between *RAS* mutational status and BV efficacy; however, this investigation was performed on small study population and only a subgroup of patients (*n* = 35) was treated with BV-containing regimes [14]. Interestingly, a recent pioneering study on 167 patients who underwent lung metastasectomy for mCRC, has reported for the first-time that for patients with *KRAS* codon 12 mutations, perioperative bevacizumab was associated with a significant improvement in both loco-regional recurrence free survival and OS [15]. These preliminary findings indicate that EGFR pathway genes mutations, and particularly *KRAS* exon 2 mutational status, could represent potential predictive markers of targeted therapy with BV and could help in selecting patients who will achieve a clinical benefit from BV administration. Moreover, recent available data, that suggest a detrimental impact of *RAS* mutations on BV efficacy, could contribute to better clarify the potential role of this anti-VEGF drug in the first-line treatment of mCRC patients with *RAS* mutated status, an issue that is still under debate [32]. However, since the heterogeneity (e.g., ethnicity, study design and statistical power, co-treatment) and the retrospective nature of the few published studies, additional larger independent and prospective trials are needed to clarify the real clinical contribution of *RAS/PIK3CA/BRAF* mutations in optimizing bevacizumab therapy prior to their introduction in the routine practice.

### 2.2. Regorafenib 

The multi-kinase inhibitor regorafenib (STIVARGA^®^), approved by the FDA in September 2012 for third-line treatment, exerts antiangiogenic activity on a broader spectrum of cellular targets downstream of the VEGF cascade, including VEGFR1, 2, and 3; fibroblast growth factor receptor (FGFR); platelet-derived growth factor receptor (PDGFR); and the proto-oncogenes rearranged during transfection (RET), tyrosine kinase with Ig and EGF homology domains (TIE-2), discoidin domain receptor 2 (DDR2), RAF-1, and BRAF [31,33]. Two large multicenter, multinational, and randomized phase III clinical trials carried out in 2013 (CORRECT trial) and 2015 (CONCUR trial) demonstrated the benefit of regorafenib in terms of OS and highlighted the existence of clinicopathological stratification factors that identify subgroups of patients likely to benefit from administration of this targeted agent: the number of metastatic sites, time from diagnosis to metastatic disease, and previous treatment with anti-VEGFR agents. However, no validated genetic predictive/prognostic markers have been identified to date [34,35]. In CORRECT and CONCUR clinical trials of 760 and 204 mCRC patients of different ethnicities randomized to receive 2:1 regorafenib and placebo, neither *KRAS* nor *BRAF* mutational status was predictive of outcome. A confirmation of these preliminary observations derived from the retrospective analysis of the CORRECT trial data set by Tabernero et al. did not find any significant association between any of the analyzed somatic variants in the *KRAS* or *PIK3CA* genes and patient outcome in terms of OS and PFS [16]. Conversely, a multicenter study (REBECCA) performed with 654 mCRC patients receiving regorafenib within a compassionate use program suggested a role of *KRAS* status as a predictor of shorter OS independent of clinicopathological factors, such as performance status, regorafenib dose, and number of metastatic sites [17]. At present, the involvement of *KRAS/PIK3CA* somatic mutations in modulating regorafenib efficacy is uncertain, and additional research is needed to clarify the real clinical contribution of EGFR pathway genes mutations in optimizing the administration of this multi-kinase inhibitor. Moreover, because regorafenib works on multiple targets, it may also expand the pharmacogenomic evaluation to consider mutations in genes encoding other proteins involved in the drug mechanism of action and adopt a combinational approach to selecting the best genetic profile for the response to treatment. A support for this hypothesis is derived from a case report describing an outstanding response to regorafenib related to the presence of the VEGFR2c.2881C > T mutation, the functional meaning of which should be further clarified [18]. These data, although of hypothesis-generating nature, surely solicit further research efforts to investigate the predictive value of molecular markers belonging to the angiogenesis pathway in regards to regorafenib efficacy.

### 2.3. Ziv-Aflibercept

The angiogenic inhibitor ziv-aflibercept (Zaltrap^®^) is a recombinant fusion protein composed of the VEGF-binding portions of the extracellular domains of receptors 1 and 2 fused with the fragment crystallizable (Fc) portion of human IgG1 able to bind VEGF (-A and -B) ligands and placental growth factor (PIGF), blocking the VEGF pathway. Its approval by the FDA and EMA in 2011 and 2012, respectively, was based on the results of the phase III VELOUR trial in which ziv-aflibercept demonstrated improved OS and PFS in association with FOLFIRI in 612 mCRC patients who had previously failed an oxaliplatin-based regimen [31,36]. To date, no validated pharmacogenomic markers associated with ziv-aflibercept effectiveness have been identified. Only one study, performed in 236 mCRC patients treated with mFOLFOX6 alone or in combination with ziv-aflibercept (phase II AFFIRM trial), assessed a panel of 96 somatic variations in *KRAS, BRAF, NRAS, PTEN, PIK3CA, EGFR, PIK3R1* and *PIK3R2* genes in order to correlate patient outcomes with genetic markers. None of the analyzed somatic mutations significantly correlated with PFS. Only a trend was observed for mutant *KRAS* tumours that exhibited a slightly worse median PFS for mFOLFOX6 compared with mFOLFOX6 plus aflibercep regimen [19]. Additional biological targets involved in the ziv-aflibercept pathways signaling will be assessed to find potential predictive markers of ziv-aflibercept efficacy. Because ziv-aflibercept acts as a trap for VEGF and PIGF ligands, mutations in genes encoding these targets and the related cascade may represent good candidate biomarkers of drug resistance, though further investigations are needed to confirm this hypothesis.

### 2.4. Ramucirumab

Ramucirumab (Cyramza^®^) is a human mAb that specifically targets VEGFR-2, inhibiting the angiogenic pathway by blocking the binding of VEGF ligands to the receptor. The FDA approved this targeted molecule in April 2015 for the second-line treatment of mCRC patients who previously received bevacizumab, oxaliplatin, and fluoropyrimidine-containing regimens. The approval was consequential to the improved survival in a double-blind, multicenter, phase III trial (RAISE) based on 1072 mCRC patients randomized 1:1 to receive ramucirumab plus FOLFIRI versus placebo plus FOLFIRI [16,20]. Due to the importance of identifying patients who can be considered good responders to ramucirumab, a pharmacogenomic analysis was performed on the RAISE trial data set, stratifying patients according to certain parameters, including *KRAS* mutational status. No significant differences were detected between the tumoral *KRAS* wild-type or mutant considering both arms in terms of survival; only a trend of longer OS was observed in *KRAS* wild-type patients compared to *KRAS* mutant patients in the ramucirumab arm [20]. A case report study of a patient treated with ramucirumab-containing therapy and carrying the *KRAS* wild-type suggested that a somatic missense mutation in the VEGFR-2 gene (p.T771R) leading to self-activation of VEGFR-2, thus stimulating angiogenesis, could contribute to acquired drug resistance [21]. Considering the recent introduction of ramucirumab into clinical practice, no other data have been published regarding genetic profiles that could predict drug effectiveness. However, given that ramucirumab exclusively targets the extracellular domain of VEGFR-2, somatic variants in *KDR*, the gene encoding VEGFR-2, could be the focus of future research to better understand the variability of the ramucirumab response.

Ramucirumab (Cyramza^®^) is a human mAb that specifically targets VEGFR-2, inhibiting the angiogenic pathway by blocking the binding of VEGF ligands to the receptor. The FDA approved this targeted molecule in April 2015 for the second-line treatment of mCRC patients who previously received bevacizumab, oxaliplatin, and fluoropyrimidine-containing regimens. The approval was consequential to the improved survival in a double-blind, multicenter, phase III trial (RAISE) based on 1072 mCRC patients randomized 1:1 to receive ramucirumab plus FOLFIRI versus placebo plus FOLFIRI [16,20]. Due to the importance of identifying patients who can be considered good responders to ramucirumab, a pharmacogenomic analysis was performed on the RAISE trial data set, stratifying patients according to certain parameters, including *KRAS* mutational status. No significant differences were detected between the tumoral *KRAS* wild-type or mutant considering both arms in terms of survival; only a trend of longer OS was observed in *KRAS* wild-type patients compared to *KRAS* mutant patients in the ramucirumab arm [20]. A case report study of a patient treated with ramucirumab-containing therapy and carrying the *KRAS* wild-type suggested that a somatic missense mutation in the VEGFR-2 gene (p.T771R) leading to self-activation of VEGFR-2, thus stimulating angiogenesis, could contribute to acquired drug resistance [21]. Considering the recent introduction of ramucirumab into clinical practice, no other data have been published regarding genetic profiles that could predict drug effectiveness. However, given that ramucirumab exclusively targets the extracellular domain of VEGFR-2, somatic variants in *KDR*, the gene encoding VEGFR-2, could be the focus of future research to better understand the variability of the ramucirumab response.

### 2.5. Cetuximab 

Cetuximab (Erbitux^®^) is a DNA recombinant mAb that binds selectively to the extracellular domain of EGFR (encoded by *HER-1*) with a 5 to 10-times higher affinity than endogenous ligands, inhibiting the EGFR pathway and tumor growth. Cetuximab was approved by the FDA in 2004 for the treatment of mCRC. Several trials have focused on the effectiveness of cetuximab. Based on the results of the two main trials (CRYSTAL and OPUS), cetuximab entered into clinical practice as first-line treatment of mCRC in *KRAS* wild-type tumors [31]. Pharmacogenomic studies aiming to optimize anti-EGFR (i.e., cetuximab, panitumumab) treatment have focused mainly on somatic variants in the RAS (i.e., *KRAS, NRAS*) and RAF (i.e., *BRAF*) pathways, generating important and clinically useful results. The tumoral *KRAS* mutational status is widely acknowledged to be a crucial determinant of the response to EGFR inhibitors (EGFR-Is); thus, *KRAS* genotyping is recommended before starting cetuximab-based therapy in order to assess the presence of potential somatic mutations that, leading to constitutive activation of the EGFR pathway, could compromise the anti-tumoral effectiveness of this targeted agent [10]. The most frequently and routinely genotyped *KRAS* mutations occur in codons 12 and 13 (exon 2). As these somatic variations have been associated with primary and acquired resistance, their evaluation is mandatory prior to treatment and suggested during treatment. Mutations in codons 61 (exon 3), 117, and 146 (exon 4) of *KRAS* are less common, yet their assessment is suggested prior to treatment, particularly in tumors with wild-type codons 12 and 13, in order to better predict the response to anti-EGFR drugs (i.e., cetuximab or panitumumab) [10,37,38]. Interestingly, studies have also focused on *KRAS* c.38G > A, a rare somatic missense mutation in codon 13 (G13D) that has been associated with a better prognosis in terms of OS and PFS and a persistent sensitivity to anti-EGFR treatment [10,24]. However, other studies were not able to replicate these associations [22,23], highlighting the need for future research to clarify the real predictive value of the G13D mutation on anti-EGFR-based treatment. As the RAF pathway is well-established as being involved in intracellular signaling for cell growth and differentiation by activating the MEK-ERK axis, *BRAF* somatic mutations, even if uncommon in CRC samples (4–15%), have promise as predictive markers of resistance to anti-EGFR therapy. In particular, the *BRAF* V600E mutation (rs113488022) in exon 15, which occurs in 8-10% of colorectal tumors and is mutually exclusive with *KRAS* mutations, was found to be associated with a more aggressive tumor phenotype, lymph node metastasis, and high microsatellite instability (MSI) [26]. This variant has also been associated with less benefit from treatment, prompting its pre-treatment evaluation [10]. To date, several meta-analyses have taken into account the mutation status within RAS and RAF family genes, confirming the association between the mutated form and a poor response to anti-EGFR mAbs (cetuximab and panitumumab) and/or acquired resistance to these targeted agents [26,27,28]. In addition, somatic mutations in additional genes downstream of the EGFR cascade have been suggested to predict the response to anti-EGFR mAbs. In particular, analysis of the RAS-RAF-MAPK and phosphoinositide-3-kinase (PI3K)-AKT-mammalian target of rapamycin (mTOR) pathways responsible for modulation of tumor proliferation, migration, and angiogenesis has highlighted the presence of some somatic alterations in the *NRAS, PIK3CA,* and *PTEN* genes that could represent a potential determinant of anti-EGFR effectiveness, especially in tumors with wild-type *KRAS* codons 12–13. Regarding the *NRAS* gene, some rare variations in codons 12, 13, 61, 117, and 146 (exons 2–4) have been related to intrinsic tumor resistance and worse ORR, PFS, and OS, recommending pre-treatment, and possibly post-treatment, genotyping [10,28]. Furthermore, mutations in exons 20 and 9 of the *PIK3CA* gene have been associated with poor ORR and shorter PFS and OS, representing additional markers for which pre-treatment evaluation could be suggested [28,29]. Tumors with non-functional PTEN (mutations and/or reduced expression) were significantly correlated with lower ORR and shorter PFS and OS [28]. In addition to the EGFR downstream cascade, evidence indicates that missense somatic variations (i.e., codon 492, S492R) in genes encoding the drug-target EGFR could be related to acquired resistance to anti-EGFR mAbs, prompting its evaluation during treatment to optimize the clinical management of patients [10]. Overall, these published data highlight that several tumor mutations in the cetuximab-related pathway could be considered in order to personalize drug administration. Currently, *KRAS* mutational status is the only biomarker routinely evaluated in the clinical setting, but its combination with additional somatic determinants of the cetuximab response and/or acquired resistance should be considered to further improve anti-EGFR administration.

### 2.6. Panitumumab

The IgG2 human mAb targeting the extracellular domain of EGFR, panitumumab (Vectibix^®^), was approved by the FDA in September 2006 for the treatment of mCRC patients who experienced disease progression after 5-FU, irinotecan, or oxaliplatin-based chemotherapy. The approval was based on data reported in a phase III open label randomized trial (20020408 trial) of 463 patients receiving panitumumab plus best supportive care (BSC) versus BSC alone [31,39]. As panitumumab targets the same pathway as cetuximab, the aforementioned literature data regarding the discovery of genetic markers predictive of cetuximab effectiveness are generally applicable for panitumumab. The response to panitumumab is strongly influenced by the constitutive activation of the RAS and RAF signaling pathways, driven by well-known somatic mutations. A meta-analysis of nine studies involving mCRC patients treated with anti-EGFR agents combined with chemotherapy highlighted an improvement in OS and PFS in patients carrying wild-type RAS compared to those harboring mutations in *KRAS* exons 3 and 4 and *NRAS* exons 2, 3, and 4 [27]. Recent large studies further confirmed the negative impact of RAS mutations on panitumumab-based therapy outcome in terms of OS and PFS. Interestingly, these data suggest the opportunity to evaluate additional *KRAS* mutations beyond the exon 2 status in order to better discern subsets of patients who could most benefit from anti-EGFR mAbs [25,30] Another meta-analysis assessing the role of RAS and RAF somatic alterations on anti-EGFR-based therapy reported a significant association between *BRAF* mutations (i.e., *BRAF* V600E) and unfavorable outcomes in terms of ORR, PFS, and OS [26].

As with cetuximab, the research on panitumumab focused on other targets in addition to RAF and RAF, such as PIK3CA and PTEN, that activate tumor cell proliferation and migration, as well as invasion and neovascularization in mCRC. A meta-analysis demonstrated that somatic variations in *PIK3CA* exons 9 and 20 are significantly associated with shorter OS. Furthermore, *PTEN* mutations and loss of expression were associated with lower ORR and poor outcome [28]. Concordantly, in another meta-analysis, mutations in both the kinase domain (exon 20) and helical domain (exon 9) of the *PIK3CA* gene correlated with shorter PFS and OS in mCRC patients with wild-type *KRAS* receiving anti-EGFR mAb, but only the *PIK3CA* rs121913279 mutation in exon 20 was associated with a significant decrease in ORR compared to wild-type exon 20 [29]. Because the response rate in wild-type *KRAS* exon 2 is 40–60%, *PIK3CA* and *PTEN* somatic variations should be considered in order to most accurately define the effectiveness of panitumumab-based therapy. Further research efforts aimed to discover biomarkers that could discriminate between cetuximab and panitumumab will be required. Recently, a somatic single point mutation in exon 12 of the EGFR (1476C > A, S468R) gene has raised some interest because it seemed to confer acquired resistance to cetuximab but not panitumumab. Structural analysis of the EGFR extracellular domain has shown that the C to A substitution, resulting in a serine to arginine replacement, results in a decreased binding affinity for cetuximab rather than panitumumab [40]. This finding could improve our understanding of the small difference in the mechanism of action of cetuximab or panitumumab, and could help in determining when panitumumab can be a solution to overcoming resistance to cetuximab.

## 3. Emerging Target Molecules in mCRC Treatment

**Figure 2:**
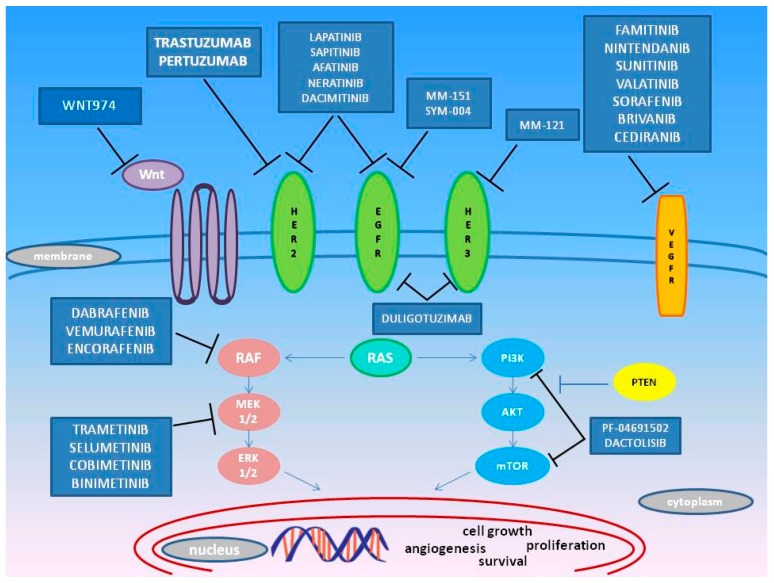
Molecular targets of recently developed agents (T arrows) designed to target additional deregulated pathways (blue arrows) in CRC (i.e., MEK/Akt, HER-2, HER-3, PI3K-mTOR, and BRAF).

Despite progress in the field of targeted therapies and the discovery of potential biomarkers to drive the choice of the most suitable molecules in advanced CRC, patients with an initial response to the therapy frequently experience disease progression [2,31]. Acquisition of resistance to anti-EGFR and anti-VEGFR agents has been associated with the occurrence of novel somatic mutations leading to up-regulation or reactivation of EGF/ VEGF downstream pathways despite the upstream blockage exercised by the targeted drug [41,42,43]. Therefore, recent research efforts have focused on characterizing the molecular features of mCRC, with particular attention on the pattern of genetic alterations, in order to better understand the mechanisms underlying the acquisition of resistance to current biological agents and to develop novel alternative targeted molecules. Below, we focus on the most relevant drugs under investigation in CRC (Figure 2).

### 3.1. HER

The human epidermal growth factor receptor (HER) family is deregulated in solid cancers, including CRC, via genetic variations, mainly somatic in nature, gene amplification, and/or protein over-expression. The stimulation of HER family components (HER-1, HER-2, HER-3, HER-4) leads to the activation of downstream signaling pathways, such as RAS/RAF/MAPK and the PI3K/AKT axis, that stimulate cell growth [10,44]. The mainstay of biological therapy in mCRC patients harboring wild-type *KRAS/NRAS* tumors is cetuximab and panitumumab therapy targeting the EGFR family (i.e., the *HER-1* gene). The development of resistance to these drugs depends on acquired tumor mutations in the extracellular domain (ECD) of EGFR, as well as members of EGFR-downstream signaling pathways (i.e., BRAF, MEK, PI3K, and AKT), that lead to an auto-activation of these crucial regulatory proteins [41,43,45]. For example, two somatic alterations in the ECD of EGFR leading to an amino acid change (G465R, G465E) near the cetuximab binding site were detected in wild-type *KRAS* tumors after cetuximab administration and suggested to be associated with acquired drug resistance [41]. Recent data highlighted that somatic variation in *HER-2* that causes HER-2 overexpression or amplification could further contribute to resistance to anti-EGFR agents [41,46]. A study of 3256 patients with advanced CRC and tumoral wild-type *KRAS* and *BRAF* detected a significant HER-2 (encoded by *ErbB2*) amplification associated with topoisomerase IIα (TOP2A) over-expression, which was suggested to be implicated in the observed disease progression despite the initial good response to anti-EGFR mAbs [46]. In light of these data, it could be hypothesized that trastuzumab (Herceptin), a mAb targeting the HER-2 receptor that is widely employed in both HER-2-positive breast and gastric cancer, could be an effective alternative for mCRC patients who experience resistance to cetuximab or panitumumab. Consistent with this suggestion, a recent case report assessed the off-label use of trastuzumab emtansine (TDM-1) in a mCRC patient that underwent rapid progression to cetuximab, likely because of the presence of HER-2 amplification. TDM-1 administration resulted in a significant improvement in the patient’s functional status and response to targeted agent, suggesting that this mAb can be used in the management of CRC [47]. Moreover, a phase II trial (HERACLES) evaluated the effectiveness of trastuzumab combined with either the reversible HER-2 inhibitor lapatinib or anti-HER-2 mAb pertuzumab in 27 mCRC patients with tumoral wild-type *KRAS* exon 2; this study reported that an objective response was achieved in 30% and complete response in 4% of the trastuzumab/lapatinib cohort, suggesting that this combination has synergic positive effectiveness in mCRC patients refractory to commonly used anti-EGFR mAbs [48]. However, a previous phase I study assessing the co-administration of pertuzumab and cetuximab in mCRC patients refractory to cetuximab therapy was not able to demonstrate any improvement in the antitumor activity for the combinatorial regimen, probably due to the overlapping adverse events that significantly affect patient management [49]. The emerging hypothesis of the combinatorial administration of HER inhibitors for mCRC treatment has encouraged research aimed at characterizing the tumor genetic background and better clarify the molecular mechanisms underlying drug resistance. A study performed on 196 formalin-fixed paraffin-embedded (FFPE) wild-type *BRAF* specimens from Caucasian patients to characterize Lynch and Lynch-like CRC, a form of hereditary nonpolyposis CRC, detected frequent mutations in the *HER-2, PIK3CA*, and *KRAS* genes, among others [50]. Regarding the *HER-2* mutational status, two variants in particular, p.L755S and p.V842I, seem to have functional implications in activating the HER-2 kinase domain, and mutated *HER*-2, together with high mismatch instability (MSI-H), was suggested to predispose an individual to higher sensibility towards trastuzumab combined with irreversible HER inhibition (i.e., aftinib, neratinib, and dacimitinib) rather than reversible HER inhibition (i.e., lapatinib and sapitinib) [50]. Preliminary findings derived from a case report characterizing a CRC sample further suggested that the simultaneous presence of *HER-2* p.L755S and *BRAF* p.N581S mutations decreases the response to trastuzumab combined with 5-FU and leucovorin administration [51]. All of these data suggest that characterization of the *HER-2* status is potentially useful for choosing more suitable targeted agents, as the administration of appropriate therapeutic molecules is one of the biggest challenges in the era of tailored therapies. Particular attention is warranted by the *HER-2* p.L755S variant, which appears to be implicated in the anti-HER-2 resistance mechanism, and to deeply understand the synergic combination effects of dual or multi HER-2 pathway blockade.

Human epidermal growth factor receptor 3 (HER-3; encoded by *ErbB3*) is an EGF family member that does not exhibit kinase activity; its activation is driven by heterodimerization with other EGF family members. As HER-3 is the main regulator of the PI3K-AKT pathway, the mAbs against EGFR and HER-2 are responsible for HER-3 de-phosphorylation and, thus, the loss of PI3K-AKT signal. This evidence suggests that HER-3 may be involved not only in the response to anti-EGFR and anti-HER-2 agents, but also in acquired resistance to these molecules; in light of the key regulatory properties assigned to HER-3, this receptor has recently become the target of some novel drugs [52]. The efficacy of the anti-HER-3 drug seribantumab (MM-121) is under investigation due to its high affinity for the ECD of HER-3 and its ability to displace heregulin, blocking the signal for PI3K/AKT activation. Heregulin is a soluble secreted growth factor that, upon binding and activation of HER-3 and HER-4 transmembrane receptor tyrosine kinases, is involved in cell proliferation, invasion, survival, and differentiation in normal and malignant tissues [53]. Currently, few preliminary data derived from both in vivo and in vitro models are available with regards to the efficacy of MM-121, and the use of this molecule for mCRC treatment is still far off [52]. In an encouraging phase I study of 12 refractory mCRC patients, the dual inhibitor (EGFR/HER-3) duligotuzimab (MEHD7945A) exhibited partial disease control and is now under investigation in a phase II trial including 120 wild-type *KRAS* mCRC patients in order to compare its efficacy in combination with FOLFIRI with respect to the cetuximab/FOLFIRI regimen [54] (NCT01652482 [55]). All of these new molecules targeting HER-3 are being developed in hope of overcoming or delaying the onset of acquired resistance to anti-EGFR therapeutics, improving the management of metastatic disease.

Other double-target molecules were developed recently with promising preliminary results. For example, Sym004, a mixture of two mAbs that target different non-overlapping epitopes of the ECD of EGFR, was developed to find an alternative strategy circumventing the ineffectiveness of and/or acquired resistance to cetuximab and panitumumab caused by EGFR-ECD mutations. Preclinical studies and a subsequent clinical trial in 62 patients refractory to anti-EGFR therapies demonstrated the safety of Sym004 and its efficacy in enhancing antitumor activity over acquired resistance to cetuximab. In particular, a partial response has been observed in a patient harboring the EGFR S492R mutation involved in cetuximab resistance [56], encouraging further studies to investigate the effectiveness of Sym004 in relation to RAS/EGFR mutational status (i.e., 20% of acquired mutations occur in the ECD of EGFR, beyond RAS/RAF status). The antitumor activity of Sym004 has been evaluated in vitro and in vivo in CRC tumors resistant to cetuximab. Furthermore, a patient harboring a EGFR G465R mutation was reported to benefit from treatment with this targeted agent [57].

Another interesting molecule is MM-151. Similar to Sym004, MM-151 represents an oligoclonal therapeutic mixture of three fully humanized mAbs that bind to three different epitopes of the EGFR-ECD, potentially overcoming the resistance associated with EGFR-ECD mutations. Once again, a phase I clinical trial showed that this multi-target approach could represent an optimal and effective strategy for the treatment of mCRC refractory to anti-EGFR inhibitors [58,59]. Currently, another phase I-II trial is ongoing with the aim to confirm the efficacy of MM-151 combined with ONIVYDE_®_ (irinotecan liposome injection) in mCRC, and preliminary results are expected at the end of 2018 (NCT 02785068 [55]).

### 3.2. MEK

The MAPK/ERK axis is a pathway involved in regulation of the cell cycle in response to an upstream RAS and RAF activating signal. As it is well established that RAS/RAF mutations occur during treatment with anti-EGFR mAbs and represent a mechanism of acquired resistance, the combination of EGFR therapy with a MAPK kinase (MEK) inhibitor may be a promising strategy to counteract the loss of effectiveness of cetuximab or panitumumab during mCRC treatment. A phase I study of 33 *KRAS*-mutated mCRC patients receiving selumetinib (AZD6244), a MEK1/2 inhibitor, plus cetuximab showed that the co-administered therapeutics were well tolerated and resulted in antitumor activity [60] (NCT 01287130 [55]). To better understand the role of RAS status in relation to MEK/EGFR double blockage, an ongoing phase Ib/II trial is assessing the combination of panitumumab with the MEK inhibitor binimetinib (MEK162) in 90 mCRC patients stratified into four cohorts according to previous administration of anti-EGFR treatments and RAS status (NCT 01927341 [55]). The preliminary findings of this study are not currently available. Another phase I study was performed to assess the safety and effectiveness of the MEK inhibitor cobimetinib (GDC-0973) combined with the EGFR/HER-3 dual inhibitor duligotuzimab in advanced CRC harboring *KRAS* mutation. The results are expected soon (NCT 01986166 [55]). MEK blockade has also been employed in the context of multi-kinase inhibition in order to enhance the response of tumors harboring the *BRAF* V600 mutation, which negatively impacts the prognosis of mCRC patients.

BRAF inhibitor dabrafenib has been combined with the MEK inhibitor trametinib to treat 43 patients with mCRC harboring *BRAF* V600E. This combination had positive clinical results in terms of achieving a partial response (12%), complete response (2%), and stable disease (56%) [61] (NCT 01072175 [55]).

To date, no MEK inhibitor has been approved for the treatment of metastatic colorectal disease, but preliminary results from ongoing trials are encouraging and suggest potential new strategies for the management of RAS/RAF mutant tumors that represent one of the major challenges in the management of target therapies.

### 3.3. BRAF 

BRAF represents a downstream effector of the RAS pathway involved in cell growth, proliferation, and angiogenesis signal transduction and is mutated in 10–15% of metastatic colorectal disease [62]. The most common somatic variant of *BRAF* is the V600E mutation, which leads to a constitutively activated protein. The more aggressive tumor phenotype and poor drug sensitivity and prognosis linked to the *BRAF* V600E mutation have encouraged further research in recent years in order to discover alternative strategies to overcome the mechanisms underlying the resistance to anti-EGFR agents [45]. Several molecules that target BRAF are currently under investigation due to increasing interest in this protein and its predictive role in mCRC. The early agents evaluated for mCRC treatment were dabrafenib and vemurafenib based on their proven efficacy in the management of mutant *BRAF* melanoma. Corcoran et al. demonstrated a positive response to dual inhibition with dabrafenib and trametinib in a subset of 43 mCRC patients with the *BRAF* V600E mutation [61] (NCT 01072175 [55]). A case report corroborated the efficacy of this targeted agent combination successfully employed for the treatment of a *BRAF*-mutated mCRC patient that rapidly progressed after the failure of multiple systemic chemotherapy regimens [63]. The efficacy of dabrafenib administered in combination with both MEK and EGFR inhibitors (trametinib and panitumumab, respectively) is now under investigation in a phase II trial including 170 mCRC subjects with the *BRAF* V600E mutation. Preliminary results are expected after March 2018 (NCT 01750918 [55]).

Vemurafenib is a *BRAF*-V600-specific inhibitor inducing RAF/MEK/ERK pathway blockade and was approved by the FDA in 2011 for the treatment of metastatic melanoma. Its employment in CRC has been widely investigated as both a single agent and in combination regimens. Recently, a phase II pilot trial of 21 *BRAF*-mutated mCRC patients experiencing failure of at least one chemotherapy treatment and receiving vemurafenib as a single agent has not demonstrated any antitumor activity for this drug [64]. Thus, research efforts have focused on the administration of vemurafenib in combined regimens; a phase Ib trial of 19 *BRAF*-mutated mCRC patients treated with irinotecan/cetuximab combined with escalating doses of vemurafenib showed the tolerability of this therapeutic option and reported tumor growth arrest or regression [65]. The same synergic triple combination is currently under investigation in a phase II trial involving 106 mCRC patients, and the first results are expected in 2020 (NCT 02164916 [55]). Another pilot study has proposed to evaluate the effectiveness of vemurafenib plus the anti-EGFR panitumumab in *BRAF*-mutated refractory mCRC patients. This therapeutic association has demonstrated clinical activity, though modest, and good tolerability [66]. A more recent anti-BRAF agent is encorafenib (LGX818), a selective RAF kinase inhibitor that has powerful antitumor activity relative to vemurafenib and dabarfenib. A multicenter open-label phase Ib dose escalation trial assessed the efficacy of encorafenib plus cetuximab and encorafenib plus cetuximab and alpelisib, a selective PI3Kα inhibitor, in 54 *BRAF*-mutated mCRC patients, highlighting the good tolerability and high antitumor activity of encorafenib in both combinations [67]. Moreover, subsequent to the safety lead-in phase trial in which the safety and tolerability of encorafenib, binimetinib, and cetuximab was assessed, a wide multicenter randomized phase III trial involving 645 refractory *BRAF* V600E mCRC cases is ongoing to evaluate the efficacy of encorafenib combined with cetuximab with or without binimetinib (NCT 02928224 [55]). However, the results are not yet available. Other ongoing clinical trials are also testing the administration of encorafenib in *BRAF*-mutant mCRC patients refractory to treatment. For example, the effectiveness and safety of encorafenib plus cetuximab and WNT974 (an oral porcupine inhibitor in extracellular reticulum ER transduction that prevents activation of Wnt ligands, interferes with Wnt-mediated signaling, and inhibits cell growth in Wnt-driven tumors) is being assessed in a phase I/II trial including 60 mCRC patients (NCT 02278133 [55]).

A different novel line of research focuses on designing anti-BRAF molecules that can achieve a clinical benefit in *BRAF*-mutated patients who develop resistance to vemurafenib or do not respond to innovative targeted therapies. EBI-907 is a BRAF inhibitor with a broad selectivity profile, including FGFR1-3, RET, c-Kit, and PDGFRb, and exhibits cytotoxic activity in CRC cell lines refractory to vemurafenib, especially if administered in combination with anti-EGFR or anti-MEK agents [68]. BGB-283 is another dual Raf kinase/EGFR inhibitor that is currently under evaluation. This molecule has demonstrated in vitro and in vivo antitumor activity, preferably in cell lines harboring the *BRAF* V600E mutation and EGFR mutation or amplification. BGB-283 has also been suggested to overcome the lack of response to first generation BRAF inhibitors vemurafenib and dabrafenib; feedback activation of the EGF-MAPK-ERK pathway seems to be involved in this mechanism of resistance [69]. These novel targeted agents, even if requiring further data to validate their clinical effectiveness and usefulness and to define the correct setting for use, could represent a great opportunity for improving the management of mCRC patients, especially those with *BRAF* mutations, who have been considered worst responders until now.

### 3.4. PI3K

PI3K peptide is a downstream effector in the EGFR pathway and frequently mutated in human tumors. PIK3CA, which consists of a regulatory subunit (p58) and a catalytic subunit (p110), is mutated in 15–25% of CRC cases, with a prevalence of variations in exons 9 (E542K, E545K) and 20 (H1047R) and an association with constitutive activation of downstream signaling [70]. Because PIK3CA-activating mutations, especially in exon 20, have been implicated in anti-EGFR resistance by stimulating the AKT axis, the relatively high frequency of these mutations may explain, in part, why some wild-type *KRAS* mCRC patients still do not respond to mAbs directed toward EGFR [70,71]. Some promising molecules targeting PIK3 and PIK3/mTOR pathway are being evaluated for their use in advanced CRC or mCRC in order to enhance the management of mCRC patients. Buparlisib (BKM120) is an oral pan-class I PIK3 agent with demonstrated antitumor activity in lung cancer that is now under evaluation for potential clinical application in CRC. Due to the positive response in preclinical cancer models, a phase I dose escalation and expansion trial including patients with advanced solid tumors, mostly CRC, evaluated buparlisib as a single agent, demonstrating remarkable antitumor activity and an acceptable safety profile [72]. One in vitro and in vivo study evaluated the combination of buparlisib and cetuximab in *KRAS*-mutated/PI3KCA wild-type tumor cells and reported significantly enhanced antitumor activity, suggesting that buparlisib could overcome cetuximab resistance [65]. The efficacy of buparlisib in combination therapies is currently being evaluated. A recently concluded phase I study involved 17 patients with mCRC or metastatic pancreas tumor administered buparlisib plus mFOLFOX 6 (NCT 01571024 [55]). The combination of buparlisib with irinotecan has been tested in 20 advanced CRC patients in a phase I trial and the first results are expected soon (NCT 01304602 [55]). Furthermore, an ongoing phase I/II trial that will end in June 2017 is evaluating the antitumor activity of buparlisib combined with panitumumab in 22 wild-type *KRAS* mCRC patients (NCT 01591421 [55]). Another interesting molecule is alpelisib (BYL719), a selective oral inhibitor of the class I PI3K catalytic subunit p110α. Promising results emerged from the in vitro study by Fernandes et al., in which alpelisib exhibited specific PI3Kα inhibition in CRC cells harboring *KRAS* mutations, suggesting a potential alternative therapeutic approach for the management of refractory diseases and encouraging further studies to investigate this novel agent [73]. In this study, alpelisib demonstrated preliminary antitumor activity, and its employment in advanced CRC is under investigation because CRC cells with *KRAS* and *PI3K* mutations are sensitive to PI3K p110α inhibition. Currently, a phase I-II trial involving 150 *BRAF*-mutated mCRC patients is assessing the combination of encorafenib and cetuximab with or without alpelisib; preliminary findings suggest an improvement in the survival rate for patients receiving the triplet regimen (cetuximab plus encorafenib plus alpelisib), but definitive results are expected in 2018 (NCT 01719380 [55]). Another ongoing phase I trial is evaluating the combination of alpelisib with capecitabine and radiotherapy for the treatment of CRC in order to determine the maximum tolerated dose (MTD) and response profile of this regimen (NCT 02550743 [55]). Dactolisib (BEZ235), a PI3K/mTOR dual inhibitor, is another recently developed molecule that has raised interest since its synergism with the oral MEK1/2 inhibitor selumetinib. The combination of these two targeted agents has resulted in tumor growth blockade in *KRAS*- and *PIK3CA*-mutated CRC xenografts, and the antitumor effect seemed to be associated with a downregulation of matrix metallopeptidase-9 (MMP-9) and angiogenic blockade [74]. These pre-clinical results suggest that dual blockade of the PI3K and MEK pathways may overcome resistance to MEK inhibitors, providing the rationale for performing clinical trials of this combination in patients. Similarly, PF-04691502, an ATP-competitive PI3K/mTOR dual inhibitor has exhibited potent antitumor activity in a preclinical study of *PIK3CA* exon 20 mutated (H1047R) xenograft models and warrants further evaluation in CRC patients harboring *PIK3CA* mutations [75]. This preliminary evidence suggests that *PIK3CA* mutation in exon 20 (H1047R) is a possible predictive marker for stratifying populations that benefit from this dual inhibitor and guide decisions towards more suitable targeted therapy choices.

Even though several questions are still unanswered, such as the optimal drug combination, the real clinical effectiveness, and the existence of potential biomarkers improving the choice of patients that could benefit from PI3K inhibitors, the development of agents targeting this pathway is moving forward.

### 3.5. VEGFR

VEGF-related signaling plays an essential role in the neovascularization process and the tumor cell growth, development, and migration. Several antiangiogenic agents have been approved for the treatment of advanced colorectal disease (i.e., bevacizumab, aflibercept, regorafenib, and ramucirumab), and several are still under investigation. Efforts to find novel molecules targeting the VEGFR pathway have become necessary because most cases of metastatic disease develop drug resistance by adopting alternative pathway compensation mechanisms (i.e., FGF- and PDGF-related signaling activation) [76,77]. Famitinib is a multi-tyrosine kinase inhibitor that targets VEGFR-2 and -3, PDGFR, stem cell factor receptor, FMS-like tyrosine kinase receptor, and the proto-oncogene RET. In a phase II trial involving 154 mCRC patients who failed second-line or later therapy, famitinib was well tolerated and improved PFS and the disease control rate (DCR) [78] (NCT 01762293 [55]).

The validation of these promising results is still ongoing in the tFACT phase III trial, which includes 540 advanced CRC patients and ends in July 2017 (NCT 02390947 [55]). Nintedanib is a novel triple inhibitor targeting VEGFR-1, -2, and -3, FGFR, and PDGF receptors in a balanced manner, and has been evaluated for the treatment of mCRC patients as both a single agent and in combinatorial regimens. In a phase I/II trial, nintedanib has not demonstrated any superiority in combination with mFOLFOX6 compared to bevacizumab plus mFOLFOX6 [79]. The randomized LUME-Colon 1 phase III trial was designed to evaluate the efficacy of nintedanib monotherapy in terms of PFS and OS. This trial involving 764 mCRC patients refractory to multi-drug treatment was completed in 2016 and results are now expected (NCT 02149108 [55,80]). Conversely, data regarding the development of small molecule tyrosine kinase inhibitors have been quite negative until now. Inhibitors of the VEGFR pathway, such as valatinib, brivanib, cediranib, sunitinib, and sorafenib, failed to improve patient outcomes when administered in combination with other chemotherapeutics [54]. For example, vatalinib, an oral ATP-binding VEGFR inhibitor, did not exhibit any efficacy in terms of survival rate, despite a good safety profile. In two phase III trials in which this agent was combined with oxaliplatin-based chemotherapies [81,82]. Moreover, two multi-tyrosine kinase inhibitors, sunitinib and vandetanib, have not shown any particular survival benefit in mCRC patients when administered in combination therapies in phase III and II trials, respectively [83]. Similarly, sorafenib, an antiangiogenic multi-kinase inhibitor used to treat hepatocarcinoma with positive results, has demonstrated conflicting results when used for CRC. Even though sorafenib administered in combination with irinotecan was associated with a positive response in an early phase I/II trials, the subsequent phase IIb trial evaluating sorafenib plus mFOLFOX6 has not been able to replicate this clinical correlation [84,85].

Currently, several approved anti-VEGFR agents are available for the first-line or later treatment of mCRC as single agents or in combination therapies. However, the development of acquired resistance still remains a clinical challenge. Thus, the development of new VEGFR multi-kinase inhibitors, in addition to the aforementioned novel molecules targeting pathways downstream of EGFR and VEGFR, is the main goal for future research efforts.

## 4. Conclusions and Future Directions

Patients with mCRC will benefit from the discovery and validation of predictive and prognostic biomarkers of primary tumor sensitivity to targeted agents, as well as early markers of tumor progression that could anticipate currently used clinical tools. Recent studies investigating genetic aberrations that accumulate at the tumor level and are related to acquired resistance to pharmacological treatment in CRC suggested that the study of circulating tumor DNA (ctDNA) could be a helpful source of information for a dynamic study of tumor genomic assets providing a non-invasive tool to overcome the problem of tumor heterogeneity and limit invasive tumor biopsies [86,87]. ctDNA is released by tumor cells in the circulation by multiple mechanisms (i.e., cell necrosis, cell apoptosis, or secretion). Cancer-associated genetic alterations, such as point mutations, copy number variations, chromosomal rearrangements, and methylation patterns, can be detected in ctDNA [88,89]. Genetic alterations detectable in the ctDNA, such as mutations in the RAS family of genes, precedes the clinical diagnosis of tumor progression due to secondary tumor resistance to a cetuximab-based treatment by 10 months [87]. Quick advancement in the technological field has enabled the detection of mutant DNA clones with very high sensitivity in plasma when a high normal DNA background is present. The use of highly sensitive digital genomic technologies, such as BEAMing (beads emulsion, amplification, and magnetic) or digital droplet PCR, dramatically increased the investigation of ctDNA in cancer patients. Digital genomic technologies allowed the detection and enumeration of rare variant alleles in complex mixtures of DNA [86,90,91], improving ctDNA research and leading to the publication of the first exploratory studies of the application of quantitative and qualitative analyses of ctDNA in the study of advanced CRC resistance and early treatment response [92,93,94]. The possibility of sequencing the whole exome using circulating free DNA and monitoring treatment outcomes could be an opportunity to detect new genetic markers of tumor resistance to anti-cancer agents and drive the discovery of candidate targets for new, effective anti-cancer drugs [95].

The so-called liquid biopsy will be the future scenario of pharmacogenomic diagnostics and research in cancer treatment. The possibility of recovering tumor DNA at diagnosis and at different treatment and disease stages without an invasive biopsy is a great opportunity for real-time monitoring of patient outcomes and early detection of drug resistance [96].

The evidence provided in this review highlighted that tumor somatic genomic information will help in achieving the goal of successfully stratifying patients into subgroups according to tumor sensitivity to specific treatments [97] (Table 2). Moreover, the molecular and immune classification of CRC provides a new scenario for precision medicine, highlighting innovative prognostic and predictive factors for chemo and immunotherapies. In particular, the classification of six CRC subtypes according to the combined analysis of gene expression profiles allowed the prediction of a differential response to cetuximab and differential sensitivity to FOLFIRI regimens in both adjuvant and metastatic settings [98]. More recently, the so-called immunoscore and tumor immune infiltration emerged as the best CRC patient classifiers according to prognosis and risk of tumor recurrence [99].

Investigation of the relationship between patients’ immune assets and therapeutic outcomes and the search for biomarkers for a rational use of immunotherapy agents, such as checkpoint inhibitors, will lead the field of mCRC treatment and cure in the next few years. Advancements in genomic sequencing have resulted in an increased understanding of the genomic landscape of CRC and already been demonstrated to be helpful in guiding immunotherapy, providing evidence of the importance of evaluating the number and type of alterations in the tumor genome for precision immunotherapy [98,101].

In conclusion, even though the introduction of new targeted agents in combination with conventional chemotherapy improved the treatment of CRC in the last few years, acquired tumor resistance to treatment is still a major challenge. The occurrence of somatic mutations in the RAS/RAF/MAPK and PI3K/PTEN/AKT pathways in tumors represent a major challenge for mCRC treatment with new biological agents. The pharmacogenomic research could help identify individual genetic features related to drug sensitivity and allow better patient stratification, leading to successful personalized medicine for cancer.

## Figures and Tables

**Table 1:** Summary of published works on somatic mutations and efficacy of approved targeted agents.

Gene	Rs Code	Nucleotide Change and/or Location	Therapy	Setting	Patients Population	Ethnicity	Clinical End-Points	Main Effect	Citation
**Bevacizumab**									
*KRAS*	n.a.	G12A/V (exon 2)	BV-based therapy (combination with FOLFOX, FOLFIRI, FUFA, XELOX, XELIRI, XELODA, CAMPTO or OXALIPLATIN)	Fisrt-line and greater	404 mCRC	Caucasian	PFS OS	At the multivariable analysis KRAS G12V and G12A mutations showed a lower PSF (HR = 2.18, *p* < 0.001) and OS (HR = 2.58, *p* < 0.001).	[11]
*KRAS*	n.a.	codons 12, 13 (exon 2) 59, 61 (exon 3) 117 and 146 (exon 4)	FOLFOXIRI plus BV vs FOLFIRI plus BV (TRIBE)	First-line	508 mCRC	Caucasian	OS PFS	RAS (HR = 1.49) and BRAF mutation (HR = 2.79) subgroups have shorter median OS compared with RAS/ BRAF wt subgroup (*p* < 0.0001) RAS (HR = 1.23) and BRAF (HR = 2.27) mutation subgroups have shorter median PFS compared with RAS and BRAF wt subgroup (*p* = 0.002) Comparing the two regimes, treatment effect was not significantly different across molecular subgroups	[12]
*NRAS*	n.a.	codons 12, 13 (exon 2) 59, 61 (exon 3) 117 and 146 (exon 4)							
*BRAF*	rs113488022	NM_004333.4:c.1799T > A (V600E)							
*KRAS*	n.a.	codons 12, 13 (exon 2) 61 (exon 3) and 146 (exon 4)	FOLFOX6 or CapeOX or FOLFIRI plus BV	First-line	90 mCRC	Japanese	ORR PFS	Even if not statistically significant, ORR was higher for patients with wt tumors (64.3%) compared to those with tumors that were only wt with respect to KRAS exon 2 (54.8%); the differences in ORR between patients with wt and mutant-type tumors were greater when considering only KRAS exon 2 mutations (6.8%) rather than RAS/PIK3CA/BRAF mutations (18.4%). At the multivariate analysis liver metastasis, unresectable primary tumor, RAS and BRAF tumor mutations resulted predictive for early progression.	[13]
*NRAS*	n.a.	codons 12, 13 (exon 2) and 61 (exon 3)							
*BRAF*	rs113488022	NM_004333.4:c.1799T > A (V600E)							
*PIK3CA*	n.a.	codons 542, 545, 546 (exon 9) and 1047 (exon 20)							
*KRAS*	n.a.	codons 12, 13 (exon 2) 59, 61 (exon 3) 117 and 146 (exon 4)	FOLFOX or XELOX plus BV vs. FOLFOX or XELOX	First-line	93 mCRC	Caucasian	ORR PFS OS	RAS (KRAS or NRAS) mutations are not a prognostic marker for RR, PFS and OS	[14]
*NRAS*	n.a.	codons 12, 13 (exon 2) 59, 61 (exon 3) 117 and 146 (exon 4)							
*KRAS*	n.a.	codons 12, 13	5-FU alone or in combination with oxaliplatin (FOLFOX/XELOX) and/or irinotecan (FOLFIRI/FOLFOXIRI) ± BV	Preoperative chemotherapy	167 mCRC underwent pulmonary metastasectomy	Caucasian	LRRFS OS	For patients with KRAS exon 2 codon 12 mutations , perioperative BV was associated with a significant improvement in both LRRFS (*p* < 0.001) and OS (*p* < 0.001)	[15]
*BRAF*	rs113488022	NM_004333.4:c.1799T > A (V600E)							
**Regorafenib**									
*KRAS*	rs121913529	NM_004985.4:c.35G > A (G12D)	Regorafenib plus BSC vs. placebo plus BSC (phase III CORRECT trial)	Salvage-line CT	760 mCRC (505 regorafenib arm vs. 253 placebo arm)	Caucasian, Asian, North American, Australian	OS PFS	PFS and OS were higher in the Regorafenib arm irrespective of KRAS and PIK3CA mutational status.	[16]
		NM_004985.4:c.35G > T (G12V)							
		NM_004985.4:c.35G > C (G12A)							
	rs122193530	NM_004985.4:c.34G > T (G12C)							
		NM_004985.4:c.34G > A (G12S)							
		NM_004985.4:c.34G > C (G12R)							
	rs112445441	NM_004985.4:c.38G > A (G13D)							
	rs17851045	NM_004985.4:c.183A > C (Q61H)							
	rs121913527	NM_004985.4:c.436G > A (A146T)							
*PIK3CA*	rs121913273	NM_006218.3:c.1624G > A (E542K)							
	rs104886003	NM_006218.3:c.1633G > A (E545K)							
	rs121913274	NM_006218.3:c.1634A > G (E545G)							
	rs121913279	NM_006218.3:c.3140A > G (H1047R)							
		NM_006218.3:c.3140A > T (H1047L)							
	rs121913281	NM_006218.3:c.3139C > T (H1047Y)							
*KRAS*	n.a.	Exons 2,3 and 4	Regorafenib (REBECCA observational trial)	French compassionate program	654 mCRC	Mostly caucasian	OS	In the multivariate analysis KRAS mutations were associated with shorter OS (HR:1.25; *p* = 0.016) in an independently manner from PS, number of metastatic sites and time of initial diagnosis	[17]
*KDR*	rs80338758	NM_005120.2:c.2881C > T (R961W)	Regorafenib monotherapy	Second-line	1 case report mCRC	American	RR	The patient with KDR c.2881C > T mutation experienced excellent tolerance and response to Regorafenib administration.	[18]
Aflibercept									
*KRAS*	n.a.	9 mutation in codons 12, 13 (Exon 2)	Ziv-aflibercept plus mFOLFOX6 vs mFOLFOX6 (phase II AFFIRM trial)	First-line	93 mCRC (47 treated with Aflibercept)	Asian/oriental, Black, Caucasian/white	PFS (primary endpoint)	Only patients with mutations in KRAS showed a not statistically significant trend to worse PFS when treated with mFOLFOX6 rather than mFOLFOX6 plus Aflibercept. Mutations in KRAS, NRAS and BRAF did not influenced PFS in both arms.	[19]
	n.a.	6 mutation in codon 61 (Exon 3)							
*BRAF*	rs113488022	NM_004333.4:c.1799T > A (V600E)							
	rs121913338	NM_004333.4:c.1781A > G (D594G)							
*NRAS*	n.a.	12 mutations in codons 12,13 (exon 2)							
	n.a.	5 mutations in codon 61 (exon 3)							
*PTEN*	n.a.	2 deletions (codons 267,323)							
	n.a.	5 mutations (codons 85, 173, 233, 130)							
*PIK3CA*	n.a.	22 mutations (codons 345,38,420,539,542,545,546,88,901,1043,1047,1049,106,118)							
*PIK3R2*	n.a.	C1546G > A(R345Q)							
*PIK3R1*	n.a.	17 mutations (codons 162,285,348,358,376,455,461,527,543,564,565,574,576,642,649,666,682) 5 insertions (codons 376,448,459,562–563,668–669) 2 deletions (codons 447,601)							
*EGFR*	n.a.	6 mutations (codons 289,858,719,790)2 deletions (codons 746–750, 746–750)							
**Ramucirumab**									
*KRAS*	n.a.	Codon 12,13 (Exon 2)	RAM plus FOLFIRI vs. placebo plus FOLFIRI (RAISE trial)	Second-line	1072 mCRC	mostly White and Asian	OS	KRAS wt patients showed a trend to longer OS (HR = 0.82, *p* = 0.049) in RAM-arm compared to placebo arm; KRAS mutated patients showed a trend to longer OS (HR = 0.89, *p* = 0.263) in RAM-arm compared to placebo arm.	[20]
*KDR*	n.a.	c.2312C > G (T771R)	RAM plus CTX plus CPT11	Second-line	1 case report of mCRC KRAS wt	American	n.a.	c.2312C > G variant is probably an activating mutation in response to Ramucirumab treatment	[21]
**Cetuximab**									
*KRAS*	rs112445441	NM_004985.4: c.38G > A (G13D)	CTX vs. CTX plus CPT11 (phase II trial)	Second-line and greater	29 mCRC	Japanese	PFS OS	In the KRAS G13D mutant subgroup, PFS and OS were not statistically different between the two arms. CTX effectiveness was similar in KRAS wt and KRAS G13D mutated	[22]
*KRAS*	rs112445441	NM_004985.4:c.38G > A (G13D)	CTX vs. CTX plus CPT11 (phase II trial ICECREAM)	Second-line	50 mCRC quadruple RAS wt and 50 mCRC KRAS G13D mutated	Australian	PFS	Efficacy of CTX administered alone or in combinatorial regimens was similar in quadruple RAS wt (KRAS, NRAS, BRAF and PIK3CA wt) and in G13D KRAS mutated	[23]
	n.a.	Exons 3,4							
*NRAS*	n.a.	Exons 2,3,4							
*BRAF*	n.a.	Exon 15							
*PIK3CA*	n.a.	Exons 9,20							
*KRAS*	rs112445441	NM_004985.4:c.38G>A (G13D)	CTX	n.a.	98 mCRC	Japanese	OS PFS	At the multivariate analysis there was a trend to better PFS (HR = 0.29; *p* = 0.07) in KRAS G13D mutated patients compared to other KRAS mutations.	[24]
**Panitumumab**									
*KRAS*	n.a.	codons 12 and 13 (exon 2)	PAN plus BSC vs. BSC (Phase III trial)	Second line and greater	377 mCRC KRAS exon 2 wt	Asian Caucasian and other ethnicities	OS PFS	OS was significantly longer in PAN arm in both wt KRAS exon 2 (HR = 0.73, *p* = 0.0096) subgroup and wt RAS subgroup (HR = 0.70, *p* = 0.0135). PFS was significantly longer in PAN arm in both wt KRAS exon 2 (HR = 0.51, *p* < 0.0001) and wt RAS subgroup (HR=0.46, *p* < 0.0001).	[25]
	n.a.	codons 59 and 61 (exon 3)							
	n.a.	codons 117 and 146 (exon 4)							
*NRAS*	n.a.	codons 12 and 13 (exon 2)							
	n.a.	codons 59 and 61 (exon 3)							
	n.a.	codons 117 and 146 (exon 4)							
*KRAS*	n.a.	Exons 2,3,4	PAN or CTX plus CT PAN or CTX plus BSC (Meta-analysis of 10 studies)	6 first-line 2 second-line 2 BSC	463 CRC KRAS wt	n.a.	OS PFS ORR	EGFR-Is combined with chemotherapy do not significantly increase OS (HR: 0.91 *p* = 0.63), PFS (HR: 0.88 *p* = 0.33) and ORR (RR = 1.31 *p* = 0.25) in patients with BRAF mutated CRC.	[26]
*NRAS*	n.a.	Exons 2,3,4							
*BRAF*	rs113488022	NM_004333.4:c.1799T > A (V600E)							
*KRAS*	n.a.	codons 12 and 13 (exon 2)	PAN or CTX plus 5-FU, CPT11, Oxaliplatin based CT or BSC (Meta-analysis of 9 studies )	6 first-line 2 second-line 1 third-line (BSC)	5948 mCRC	n.a.	PFS OS	EGFR-Is efficacy was found to be significantly superior in terms of PFS (HR=0.60, *p* < 0.001) and OS (HR = 0.72, *p* = 0.008) in all RAS wt patients compared with RAS mutated patients (KRAS exon 3 and 4 and NRAS exon 2,3 and 4) No difference in terms of both PFS and OS was found between KRAS exon 2 mutated and new RAS mutated subgroups.	[27]
		codons 59 and 61 (exon 3)							
		codons 117 and 146 (exon 4)							
*NRAS*	n.a.	codons 12 and 13 (exon 2)							
		codons 59 and 61 (exon 3)							
		codons 117 and 146 (exon 4)							
*KRAS*	n.a.	codons 59 and 61 (exon 3)	PAN or CTX plus CT (Meta-analysis: 22 studies)	first-line and greater	2395 mCRC KRAS exon 2 wt	Caucasian, American, Asian, African, Australian	ORR PFS OS	From 5 studies: KRAS exons 3 and 4 mutations were significantly correlated with worse ORR (OR = 0.26) and shorter PFS (HR = 2.19) and OS (HR = 1.78) From 3 studies: NRAS exons 2,3 and 4 mutations showed a trend towards poor ORR (OR = 0.23), and significant worse PFS (HR = 2.30) and OS (HR = 1.85) From 17 studies: BRAF mutations were correlated with significant worse ORR (OR = 0.29) and shorter PFS (HR = 2.95) and OS (HR = 2.52) From 6 studies: PIK3CA exons 9 and 20 mutations were significant predictors of poor ORR (OR = 0.39) and were significantly associated with shorter OS (HR = 1.43); only a trend for worse PFS was detected. From 5 studies of primary tumor: PTEN mutations showed non-significant effect on ORR whereas PTEN mutations and/or reduced expression were significantly correlated with lower ORR (OR = 0.41) and shorter PFS (PFS = 2.6) and OS (HR = 1.77)	[28]
	n.a.	codons 117 and 146 (exon 4)							
*NRAS*	n.a.	codons 12 and 13 (exon 2)							
	n.a.	codons 59 and 61 (exon 3)							
	n.a.	codons 117 and 146 (exon 4)							
*BRAF*	rs113488022	NM_004333.4:c.1799T > A (V600E)							
*PIK3CA*	n.a.	exons 1,2,9,10,20							
*PTEN*	n.a.	exons 1,2,3,4,5,6,7,8,9							
*PIK3CA*	rs121913279	NM_006218.3: c.3140A > G (H1047R)	anti-EGFR mAb (Meta-analysis: 11 studies)	n.a.	864 mCRC KRAS wt patients	n.a.	ORR PFS OS	All PIK3CA mutations were associated with overall reduced ORR (OR = 0.42 , *p* = 0.003) and the result remained significant considering only exon 20 mutated subset (OR = 0.21, *p* = 0.04) PIK3CA mutations were associated with shorter PFS (HR = 1.54, *p* = 0.006) and OS (HR = 1.4 *p* = 0.036)	[29]
		NM_006218.3: c.3140A > T (H1047L)							
	rs104886003	NM_006218.3: c.1633G > A (E545K)							
	n.a.	codon 542 (exon 9)							
*KRAS*	n.a.	exon 3 and 4	FOLFIRI plus PAN vs. FOLFIRI (phase III trial) NCT0039183	second-line	1186 mCRC KRAS wt exon 2	n.a.	PFS OS ORR	PFS was significantly longer in the PAN-arm with respect to both the KRAS exon 2 wt patients (HR = 0.73, *p* = 0.004) and all RAS wt patients (HR = 0.70, *p* = 0.007); in the same analysis there was a trend to longer OS in the PAN-arm	[30]
*NRAS*	n.a.	exon 2,3 and 4							

Abbreviations: 5-FU, 5-fluorouracil; BSC, best supportive care; BV, bevacizumab; CAMPTO, irinotecan; CapeOX, capecitabin and oxaliplatin; CPT11, irinotecan; CT, chemotherapy; CTX, cetuximab; EGFR-Is, epidermal growth factor receptor inhibitors; FOLFIRI, 5-fluorouracil leucovorin and irinotecan; FOLFOX, 5-fluorouracil leucovorin and oxaliplatin; FOLFOXIRI, 5-fluorouracil leucovorin oxaliplatin and irinotecan; FUFA, 5-fluorouracil and folinic acid; HR, hazard ratio; LRRFS, loco-regional recurrence free survival; mCRC, metastatic colorectal cancer; mFOLFOX, modified FOLFOX regimen; n.a., not available; NCT, number clinical trial; OR, odd ratio; ORR, objective response rate; OS, overall survival; PAN, panitumumab; PFS, progression free survival; PS, performance status; RAM, ramucirumab; RR, response rate; WT, wild-type; XELIRI, capecitabin and irinotecan; XELODA, capecitabin; XELOX, capecitabin and oxalaplatin.

**Table 2:** Pharmacogenomic value of the most studied genetic markers.

Targeted Agent	Biological Target	Registration Trial for mCRC	Somatic Variant Approved or Mandatory	Somatic Variant Explorative
Bevacizumab	VEGF-A/VEGFR2	TRIBE, CAIRO2, NO16966	/	KRAS, NRAS (exon 2)
Regorafenib	VEGFR-1-2-3, FGFR, PDGFR, RET, TIE-2, DDR-2, RAF-1, BRAF	CORRECT, CONCUR	/	KRAS
Aflibercept	VEGF-A-B PIGF	VELOUR	/	/
Ramucirumab	VEGFR-2	RAISE	/	/
Cetuximab	ED-EGFR	CRYSTAL, OPUS, PRIME, NORDIC, COIN	* KRAS, NRAS, HRAS (Exons 2,3,4)	PIK3CA, PTEN (all exons)
Panitumumab	ED-EGFR	PICCOLO, 20050181 trial	* KRAS, NRAS, HRAS, EGFR (Exons 2,3,4)	PIK3CA, PTEN (all exons)

* “Table of Pharmacogenomic Biomarkers in Drug Labeling (FDA)” [100].
